# Photo-Induced Phase Transitions to Liquid Crystal Phases: Influence of the Chain Length from C_8_E_4_ to C_14_E_4_

**DOI:** 10.3390/ma2031305

**Published:** 2009-09-17

**Authors:** Marcel Petri, Gerhard Busse, Wilson Quevedo, Simone Techert

**Affiliations:** Department of Structural Dynamics of (Bio)chemical Systems, Max Planck Institute for Biophysical Chemistry, 37070 Göttingen, Germany; E-Mails: gbusse1@gwdg.de (G.B.); wqueved@gwdg.de (W.Q.)

**Keywords:** photo-induced phase-transition, time-resolved, small angle x-ray scattering (SAXS), self-assembly, entropy-driven

## Abstract

Photo-induced phase transitions are characterized by the transformation from phase A to phase B through the absorption of photons. We have investigated the mechanism of the photo-induced phase transitions of four different ternary systems C_i_E_4_/alkane (i) with n = 8, 10, 12, 14; cyclohexane/H_2_O. We were interested in understanding the effect of chain length increase on the dynamics of transformation from the microemulsion phase to the liquid crystal phase. Applying light pump (pulse)/x-ray probe (pulse) techniques, we could demonstrate that entropy and diffusion control are the driving forces for the kind of phase transition investigated.

## 1. Introduction

Tetraethyleneglycol monooctylether (C_8_E_4_), tetraethyleneglycol monodecylether (C_10_E_4_), tetraethyl-eneglycol monododecylether (C_12_E_4_) and tetraethyleneglycol monotetradecylether (C_14_E_4_) are non-ionic surfactant systems (lyotropic liquid crystalline phases), which due to the amphiphilic character of their molecules solubilize both water and oil molecules. They exhibit a variety of thermodynamic stable phases at a macroscopic length with a wide range of size domains from nanometers to micrometers [1]. Depending on temperature and concentration, the ternary systems C_i_E_4_ (with i = 8, 10, 12, 14) can form microemulsions (ME) or liquid crystal (LC) phases. These phases, which are related to the different ways that both polar and apolar regions can aggregate in space, have been studied with emphasis on their structure and stability [2]. The self-assembled aggregates have a wide range of structures like spherical micelles, cylindrical micelles, lamellar phases or bicontinuous structures - just to mention the most common ones. Microemulsions can be used as model systems to study a range of fundamental liquid state phenomena like calculation of the oil-water interfacial free energy, nucleation Ostwald ripening, solubilization kinetics, the Hofmeister effect and vesicle fusion [3].

In this study, the focus is on the mechanism of the phase transition from the microemulsion phase to the liquid crystal phase (Lα-phase) with respect to increases in chain lengths of four different nonionic surfactant systems (C_8_E_4,_ C_10_E_4_, C_12_E_4,_ C_14_E_4_). The microemulsion phase is composed of a ternary mixture of a polar solvent, a nonpolar solvent and an amphiphilic agent (C_i_E_4_). The concentration of the surfactant and the corresponding alkane have been kept constant, as well as the cyclohexane water concentration. The temperature varied in the range of 7 °C to 24 °C in order to approach the microemulsion-Lα-phase boundary. At the mesoscopic scale, the simplest approach is to consider the microemulsion phase as a collection of droplets with water and oil domains of colloidal size separated by a layer of surfactant with either micelle or inverse micelle structure. The properties of bicontinuous microemulsions, consisting of water, oil and a surfactant depend to a large extent on the bending moduli of the surfactant containing oil-water interface. These moduli can be modified through the addition of copolymers [4]. The microemulsion phase exhibits a correlation of short range order, being a less ordered phase, whereas in the Lα-phase, molecules self-assemble into aggregates with correlations of long range order (anisotropic fluid) and birefringence properties. Although many molecules that exhibit liquid crystalline phases are anisometric in their shape, the self-assembly of isometric molecules into anisotropic assemblies can result in liquid crystallinity [5]. They can be classified by their structure as nematic or smectic phases, which is the most common LC phase. They can also be distinguished by the way the phase transition appears, either by concentration (lyotropic liquid crystals) or by variation of the temperature (thermotropic liquid crystals). In this study, the LC phase is a thermotropic smectic assembly with correlation lengths which cover a range from *d* = 80 Å–130 Å. The magnitude of these lattice parameters can ideally be investigated by x-ray scattering techniques and the disturbance of the structure is related to this *d*-spacing. In this work we have induced the phase transition through the novel concept of photo-absorption. The photo-induced phase transition (PIPT) has been investigated applying the recently developed technique of time resolved small angle x-ray scattering (TR-SAXS) [6]. The phase transitions are photo-induced and based on the anti-Stokes luminescence effect [7].

**Figure 1:**
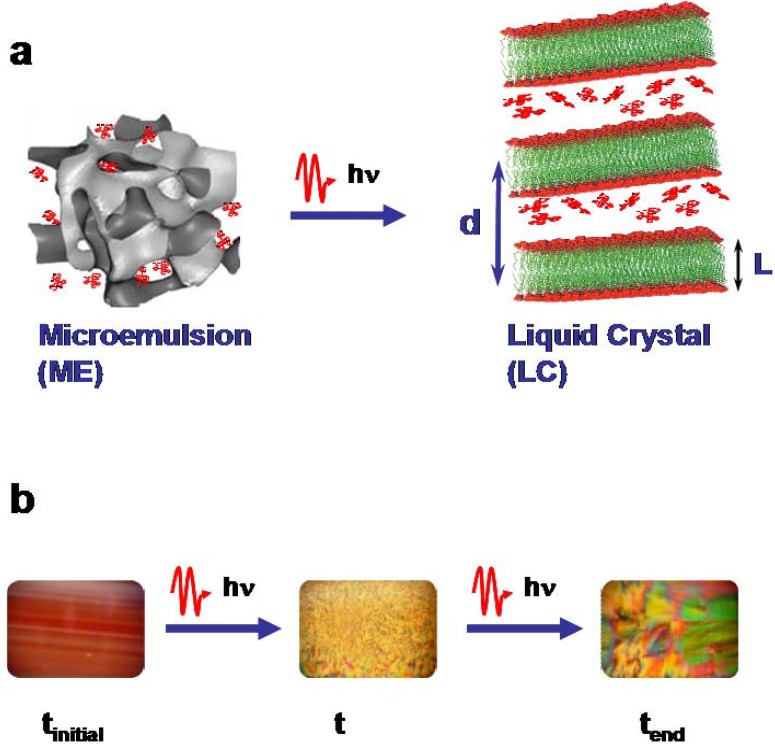
Schematic drawing of the principle of the photo-induced phase transitions (PIPT) in ternary systems from a microemulsion (ME) to a liquid crystal (LC) phase. **(a**) Structural phase transition, (**b**) optical photographs of the system C_10_E_4_ water/deca-ne/cyclohexane enriched with Rh101. Microemulsion (left), liquid crystals after partial (middle) and complete (right) transformation. The photographs of the liquid crystal phases were taken with polarizers.

In order to photo-induce the phase transitions, the solutions were photo-sensitized to make them optically active. The compound chosen was the water dye soluble rhodamin 101 (Rh101) with a quantum yield close to unity. Figure 1a schematically displays the transformation of the less-ordered microemulsion phase to the highly ordered self-organized lamellar phase (liquid crystal). Figure 1b shows on the left the optically transparent microemulsion enriched with the red laser dye Rh101 and on the right the liquid crystal as seen through crossed polarizers.

## 2. Results and Discussion

**Figure 2:**
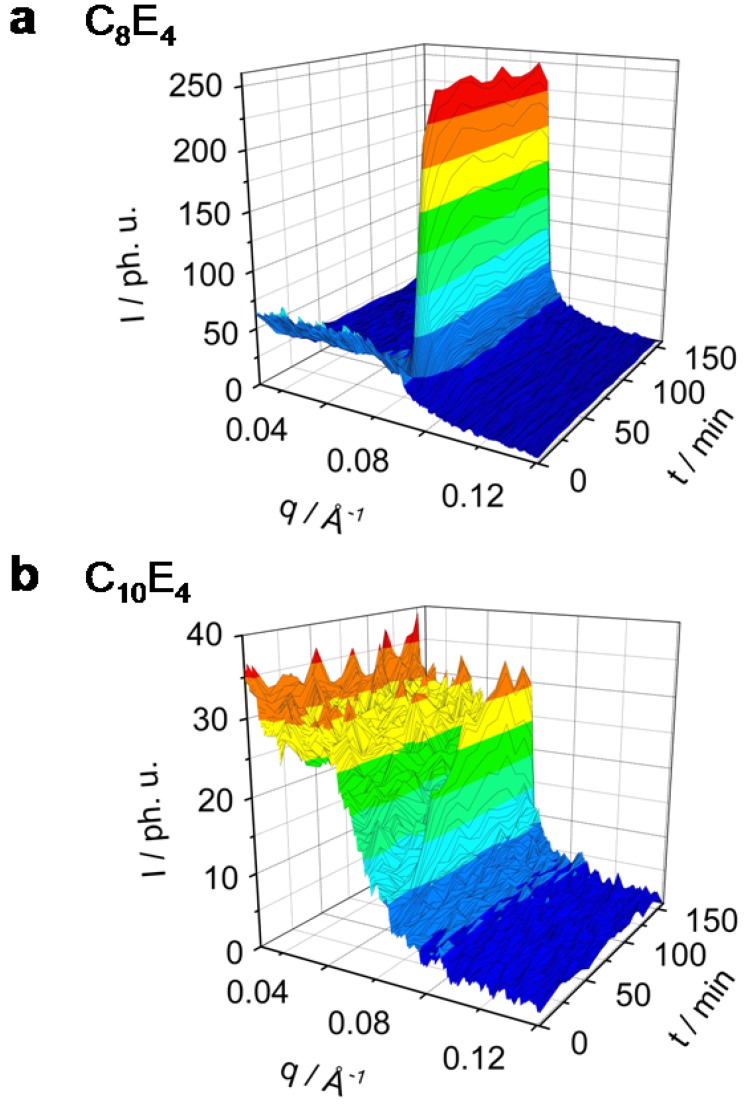
Rh101 dye-sensitized ME→LC phase transition in the system C_8_E_4_ (**a**) and C_10_E_4_ (**b**) investigated by time-resolved photo small angle x-ray scattering (TR-SAXS). C_8_E_4_ transforms completely and reaches a new equilibrium within 50 min. A significant decrease of the ME phase has been observed. C_10_E_4_ converts only partially to the LC phase on long time scales.

**Figure 3:**
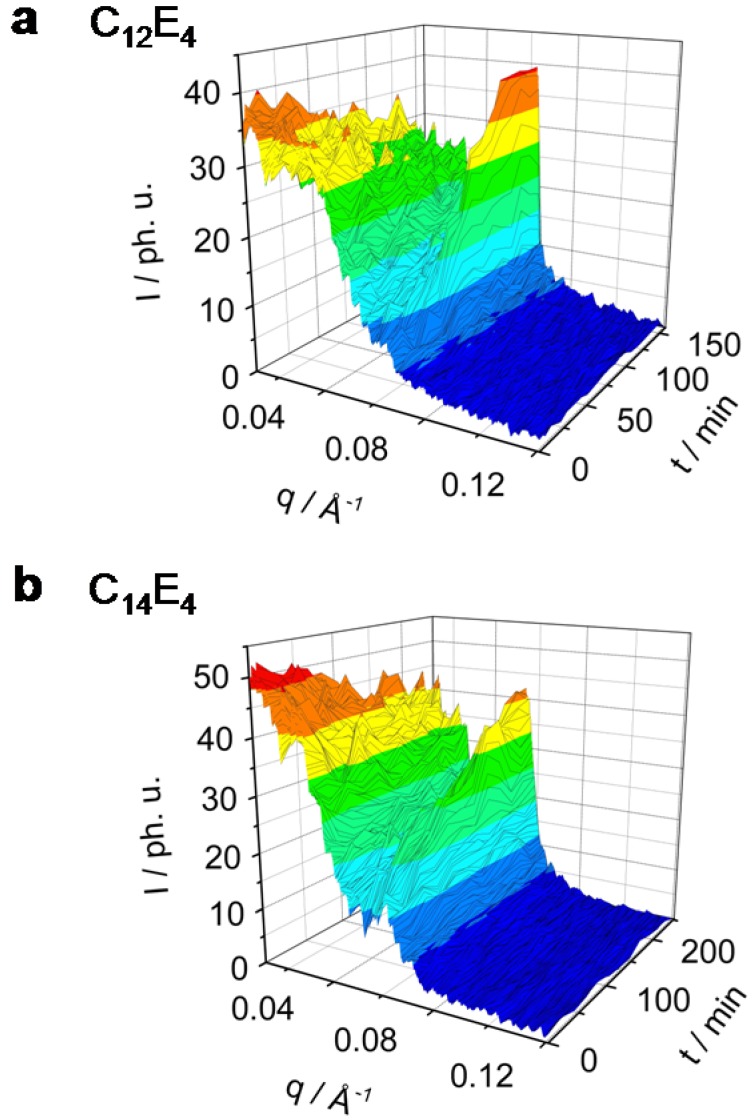
Rh101 dye-sensitized ME→LC phase transition in the system C_12_E_4_ (**a**) and C_14_E_4_ (**b**) investigated by TR-SAXS. Both systems undergo the phase transition only to less than 80%. The C_12_E_4_ has reached a new equilibrium within 100 min, C_14_E_4_ needs significantly longer (200 min).

Figure 2 and Figure 3 show in a 3-dimensional plot the photo-induced phase transition (PIPT) from the microemulsion to the liquid crystal phase (ME→LC phase transition). The ME→LC phase transition has been studied by time-resolved small angle x-ray diffraction techniques in a series of different surfactant systems ranging from C_8_E_4_ (Figure 2a) over C_10_E_4_ (Figure 2b) and C_12_E_4_ (Figure 3a) to C_14_E_4_ (Figure 3b). As has been demonstrated already, neither major intensity changes on the microemulsion peak nor the occurrence of a sharp reflection indicating the formation of a LC phase can be observed if the system is not dye-sensitized [8].

It is clearly seen from Figure 2a that the C_8_E_4_ system transforms quite easily and in large amounts, while the C_14_E_4_ system, on the other extreme, shows only a partial transformation. The photo-induction leads to an increase of the LC domains with long-range order which have been investigated as a reflection in the small angle x-ray regime. The scattering intensity maximum in the C_8_E_4_ system of the ME diffuse scatter was found to be at *q_max_* = 0.035 Å^-1^. After 25 min of illumination the diffuse scatter signal is nearly diminished and a new liquid crystal reflection at *q_max_* = 0.071 Å^-1^ has been formed. Figure 2b presents the time-dependent behaviour in the C_10_E_4_ system which shows an ME peak at *q_max_* = 0.033 Å^-1^ and the induced LC reflection at *q_max_* = 0.066 Å^-1^. The time evolution of the ME diffuse scatter and the LC reflection for the systems C_12_E_4_ and C_14_E_4_ are shown in Figure 3a and Figure 3b.

**Figure 4:**
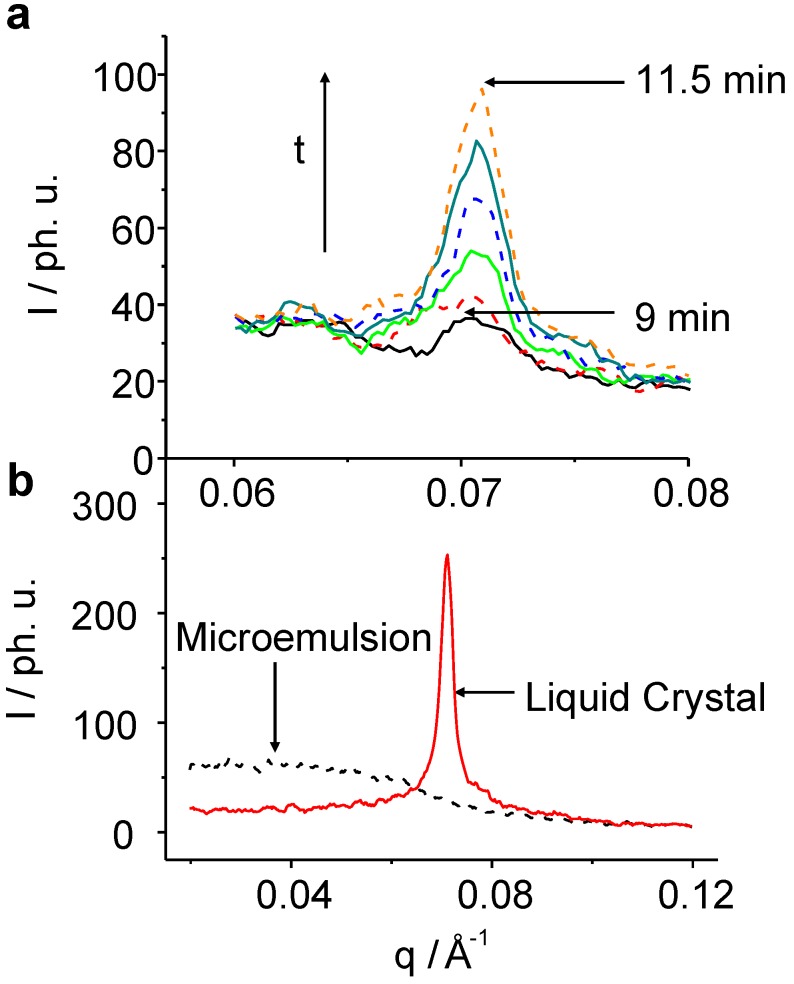
TR-SAXS pattern for the C_8_E_4_ system after the 633 nm irradiation. (**a**): Snap-shots of the creation of the LC phase with a time resolution of 30 s. The induction-time during which the nuclei reach a critical size and start to grow is about 9 min. (**b**): Pattern of the pure ME phase after 1 min of irradiation (black), and of the pure LC phase after 100 min (red).

The maximum of the C_12_E_4_ diffuse scatter reflection is at *q_max_* = 0.027 Å^-1^ and the maximum of the LC reflection is at *q_max_* = 0.065 Å^-1^. In the system which has the longest chain-length, C_14_E_4_, the maximum of the ME scatter reflection can be found at *q_max_* = 0.016 Å^-1^ and the LC reflection maximum at *q_max_* = 0.06 Å^-1^. Figure 4 shows a typical SAXS signal of the development of the liquid crystal reflection on the example of C_8_E_4_ that can be found in a similar way in all systems.

In the following, we will first choose C_8_E_4_ for describing the physical phenomena found. It takes about 9 min of irradiation without any clear signal from the LC reflection, before the peak emerges and becomes detectable among the noise. The transformation is complete after about 30 min and stable for at least 150 min before photo-degradation of the sample gives rise to enormous fluctuations of the detected signal. The observed ME diffuse scatter decrease can be quantitatively treated by means of the well known Teubner-Strey equation:

(1) $$I\;\left(t\right)\;\propto \frac{1}{a_{2}+c_{1}\left(t\right)\;q^{2}+c_{2}\left(t\right)\;q^{4}}+I_{BKG}$$

Here *I_BKG_* is the background scatter signal, *a_2_* is a time-independent scaling factor and *c_1_(t)* and *c_2_(t)* are the concentrations of the two components oil and water. The photoinduction of the phase transition is thermotropic resulting in a redistribution of the concentrations of the components oil and water within the bulk to the LC phase.

**Figure 5:**
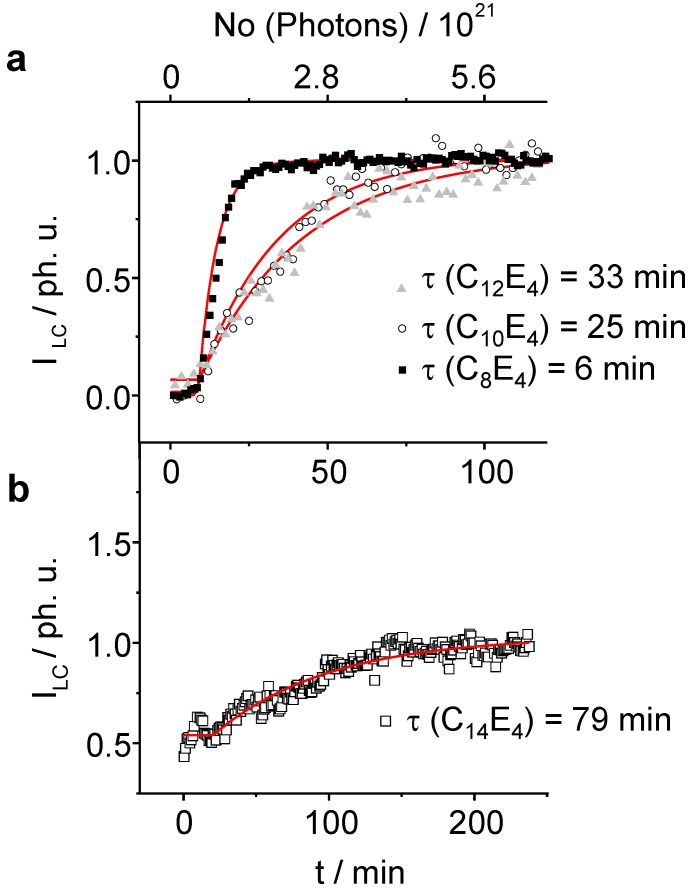
The normalized integral scattering intensity of the LC reflection exponentially increases as a function of irradiation time. The lifetime for the photo-transformation increases as the chain length of the system gets longer. (**a**): C_8_E_4_, C_10_E_4_, C_12_E_4_ (**b**): C_14_E_4_. For C_14_E_4_ the temperature was set to a value where the system was already partially transformed through thermal fluctuations leading to the creation of several LC germs. Several attempts failed to induce a PIPT from the pure ME phase.

The liquid crystal reflection increase can be described as a Gauss function with:

(2) $$I=h*\exp\left(-4\ln(2){\left(\frac{q-g}{d}\right)}^{2}\right)$$

where *I* is the scattered intensity, *h* the height of the Gauss-peak, *g* the position on the x-axis and *d* the FWHM. Levenberg-Marquard fit algorithms were applied to fit the data, to subtract the background and to extract the integral intensity of the two phases. In the time-resolved experiments, for each time point, the mass fraction of the LC and ME phases has been determined in this manner. Applying the described models, the time evolution of the whole PIPT process can be plotted as a function of time as presented in Figure 5. For reasons of clarity the intensity of the liquid crystal reflection has been normalised to one.

**Figure 6:**
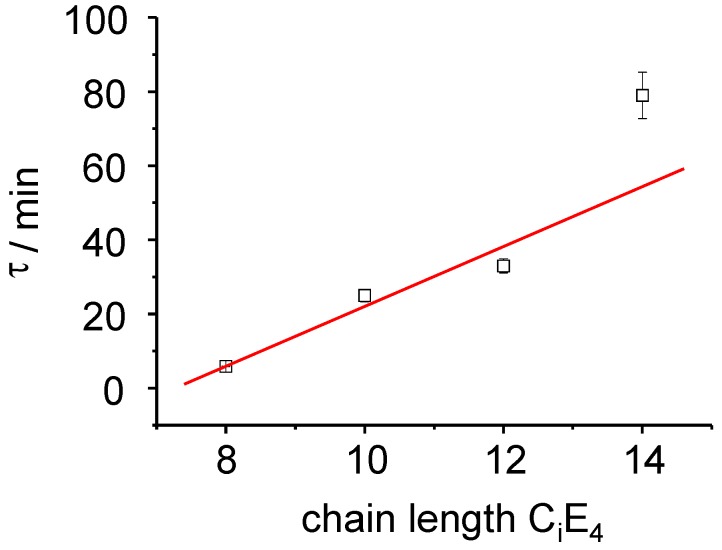
The rate constant of the PIPT exhibits a linear relationship to the chain length of the C_i_E_4_ systems as *dτ/dC_i_* = 8 min.

As already be seen in the 3-dimensional plots, the induction phase lasts for 10 minutes before the transformation begins (Figure 5a and Figure 5b). Afterwards, the integral intensity of the liquid crystal phase follows a first order kinetics. The fastest time constant has been refined to *τ _LC_* = 6 min ± 0.1 min for the C_8_E_4_ system. With increasing chain length *τ _LC_* increases for C_10_E_4_ to *τ _LC_* = 25 min ± 2 min and for C_12_E_4_ to *τ _LC_* = 33 min ± 2 min. The LC intensity increase of the C_14_E_4_ is significantly lower with *τ _LC_* = 77 min ± 5. The time constant of the ME→LC phase-transformation depends linearly on the chain length as is demonstrated in Figure 6.

**Figure 7:**
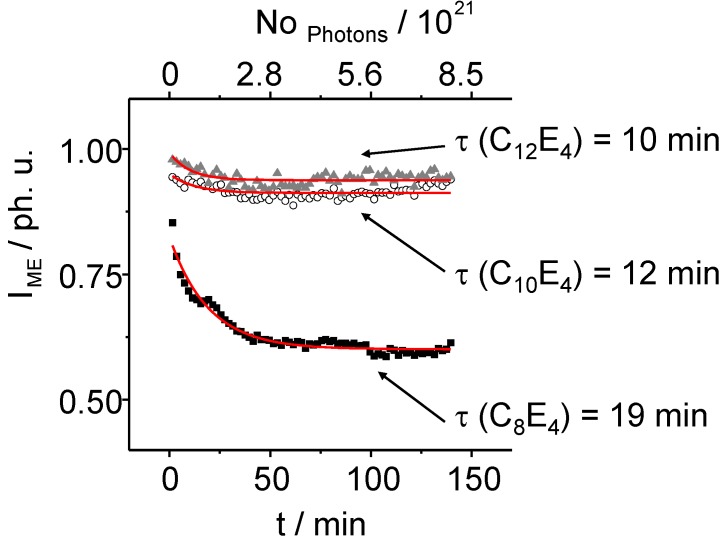
The normalized intensity of the ME diffuse scatter signal decreases exponentially. After 75 min of light illumination, the ME phase intensities decrease anymore showing the coexistence of the ME and LC phase during that time. Especially in long chain systems the PIPT ends in an imperfect state.

The intensity of the microemulsion phase also decreases monoexponentially with time. Increasing the chain length results (Figure 7) in a minor transformation of the less ordered phase to the higher ordered LC ones. While in the C_8_E_4_ system the integral intensity is reduced to 60%, only a reduction from 100% to 90% can be observed in case of the C_10_E_4_ system and to 94% in the C_12_E_4_ system. The transformation shows an inverse trend, compared to the growing time of the LC reflection.

**Figure 8:**
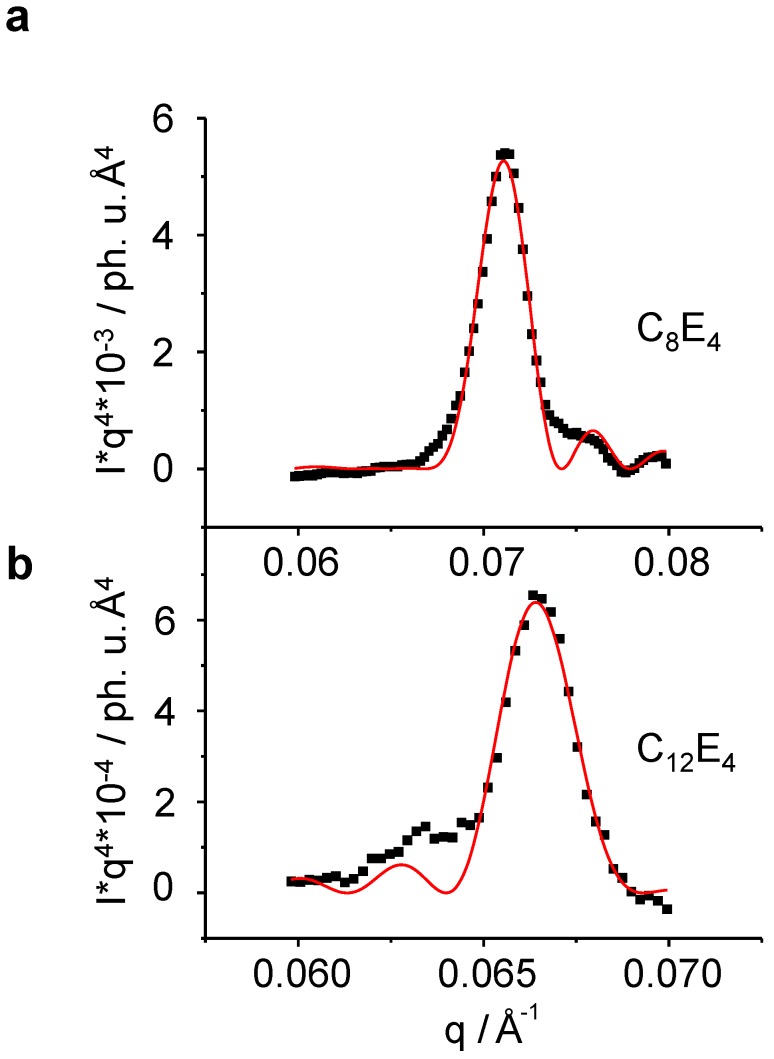
Porod fitting of the liquid crystal reflection allows for the determination of several experimental structural parameters as a function of time, such as the lamellar periodic unit *d,* the thickness of the lamella *L* and the number of lamellae *N* (Equation 3 and Figure 1). (**a**): C_8_E_4_ , (**b**): C_12_E_4_*.* The intensity of the short chain length system is significantly lower than that of the long one thus allowing for a less noisy fitting.

Time constants of *τ _ME_* = 10 min ± 3 min for C_12_E_4_, *τ _ME_* = 12 min ± 5 min for C_10_E_4_ and *τ _ME_* = 19 min ± 1 min for C_8_E_4_ have been determined. The structural analysis of the LC reflection in detail has been performed by applying the so called Porod equation. The Porod equation is a Bessel function of first order and is defined as:

(3) $$q^{4}I\;\left(q,t\right)/I^{0}\approx \sin^{2}\left(L\;\left(t\right)\;q\;/2\right)\frac{\sin^{2}\left(N\;\left(t\right)\;d\;\left(t\right)\;q/2\right)}{\sin^{2}\left(d\;\left(t\right)\;q/2\right)}$$

where the parameters are defined as *I ^0^* = 8π *N_cryst_ A* (*ρ_m_*-*ρ_p_*) *d*, *N* is the number of lamellae, *d* is the spacing, *L* is the thickness and *A* is the surface of the lamellae, *N_cryst_* are the number of (identical) liquid crystals, *ρ_m_* is the electron density of the interlayer, *ρ_p_* is the electron density of the lamella layer. Porod fitting on the LC reflection after the phototransformation is shown on the instance of C_8_E_4_ and C_12_E_4_ in Figure 8.

**Figure 9:**
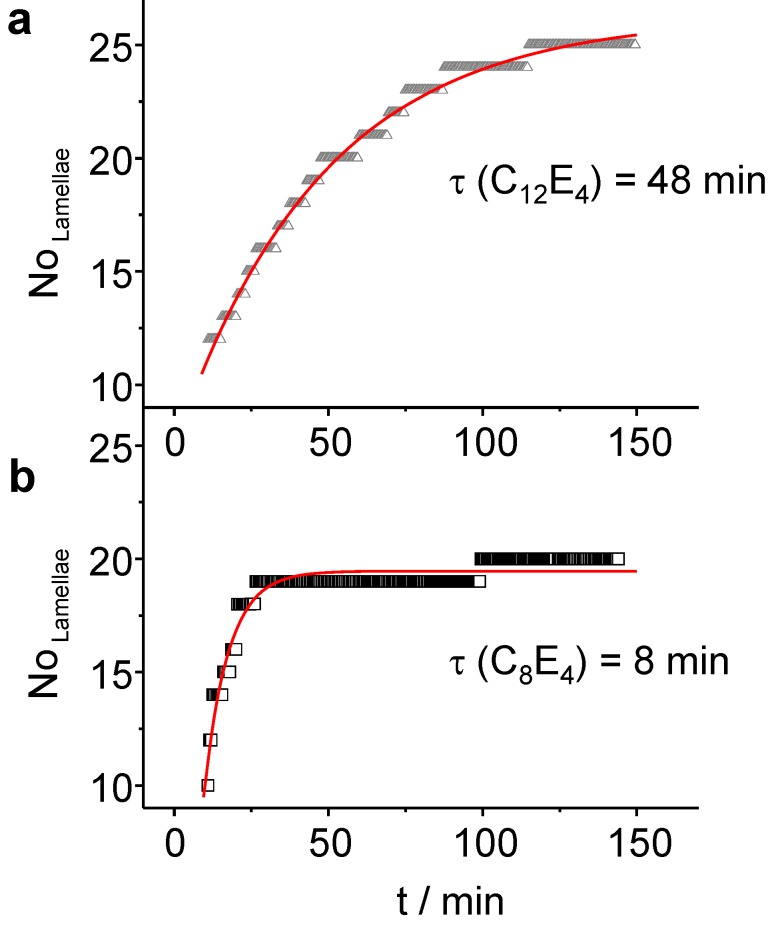
(**a**) In the course of the slow transformation (*τ* = 48 min) of C_12_E_4_, LC domains with about *N* = 25 lamellae are created. (**b**) The formation of the LC is faster in C_8_E_4_ (*τ* = 8 min) leading to a better transformation of the bulk, but with a reduced number of lamellae (*N* = 20).

For the fitting it is assumed that the electron density changes as a function of time cannot be resolved by the resolution of the scattering momentum provided by the home laboratory Kratky apparatus. Therefore these parameters (as well as all the other product parameters of *I^0 ^*) were kept constant. As time-dependent parameters *L = L(t), N = N(t)* and *d = d(t)* have been refined. Figure 9 summarises the Porod analysis for both, the C_8_E_4_ system and the C_12_E_4_ system.

For these systems, *N* = 20 (C_8_E_4_) and *N* = 25 (C_12_E_4_) lamellae have been refined. The increase follows a monoexponential law with *τ _LC_* = 7.8 min ± 0.3 min for C_8_E_4_ and *τ _LC_* = 48 min ± 1 min for C_12_E_4_. The time constants for the number of lamellae adopt values which are two times the rate constant for the LC integral intensity (Figure 6). This suggests that, after most of PIPT has been accomplished, several domains are diminishing on the benefit of other domains that are growing through the attachment of new molecules. For very long irradiation times the systems are subject to concentration fluctuations which lead at long time-scales to fluctuating intensities of the reflection. Finally, the system photo-degrades.

The distance of the lamellae decreases monoexponentially during time, indicating that the LC phase reaches a higher ordered state. It is worth to mention that this effect is dependent on the chain length. During constant illumination, the characteristic periodicity constant *d* lowers by 0.7% from 88.9 Å at the beginning of the phase-transformation to 88.3 Å when a quasi-stationary state has been reached in the C_8_E_4_ system. Liquid crystals with longer chain lengths have a larger *d*-spacing which shrinks more significantly as C_8_E_4_. C_12_E_4_ shows a decline of about 3% from 98 Å to 95 Å, for example.

The decrease of the *d*-lattice constant indicates a volume contraction and an additional ordering process of the LC system after the PIPT, which is more pronounced in systems that are more sterically hindered because of their long chain lengths.

For the further discussion on the mechanism of the ME→LC PIPT it is necessary to describe the optical excitation process in more detail. Anti-Stokes excitation of the Rh101 dye in the C_i_E_4_ microemulsion systems leads to a local decrease of temperature inside the sample which is distributed through the whole sample volume by diffusion and collision processes. The concept of Anti-Stokes excitation for extracting energy of the systems has been proved in references [9,10,11], *i.e.,* in the work of Rumbles *et al*. it has been shown that within 4 h the temperature of 0.3 mL of a solution of 10^-4^ M Rh101 in acidified ethanol (10^-2^ M HCl) has been reduced by 3 K (laser power : 350 mW). In our systems the temperature decrease has been detected to be 1K [8]. Exciting the dye with 632.8 nm in the fluorescence anti-Stokes regime leads to an excitation near to the vibrational (0-0) transition of the vibronic S_1_←S_0_ transition. The fluorescence is driven by energy transfer mechanisms based on the collision of the dye molecules first with its surrounding neighboring molecules. The energy entry to the dye molecules allows intramolecular vibrational redistribution processes as some kind of up-conversion processes in the dye molecule so that the optically resonant S_1_ state (*v* = 0) is populated from which fluorescence is possible. In a minor part of the dye molecules ordinary internal conversion processes occur or excimer like structures are formed. Via this mechanism centers of lower temperature inside the ME phase are formed. They are homogeneously distributed. The ratio of the number of incoming optical absorbed photons and the number of photo-excitable dye molecules control therefore the PIPT.

The classical theory of nucleation which also describes phase transitions or PIPTs is based on the idea that the formation of a nucleus in the crystalline phase is hindered by the surface free energy of the nucleus. With the laser power used, about 10 min are needed to create the seeds for the LC phase. Additionally, spontaneous and random ordering processes of the microemulsion take place. In accordance with the classical nucleation theory many instable germs have to come along since the nuclei have overcome the critical size for formation. The photo-induction stimulates concentration fluctuations so that the germs start to self-assemble and grow creating long range order in the bulk. By means of these processes, regions of the mixture within the capillary slowly transform up to transformation of the whole sample volume. In the general theoretical approach for the ME→LC phase transition the energy barrier that needs to be surmounted depends on the degree of undercooling. It is assumed that this transition, whose kinetics follows Arrhenius type behaviour, is of thermal character. $\Delta G^{*}$ is the activation energy and *C* the Arrhenius constant:

(4) $$k=\frac{1}{\tau }=C•\exp\left(-\frac{\Delta G^{*}}{k_{B}T}\right)$$

The free enthalpy $\Delta G^{*}$ which is a free energy, is defined as $\Delta G^{*}=\Delta H^{*}-T\Delta S^{*}$, so that the transition entropy of the system can be derived from the kinetics relation to:

(5) $$\ln\left(\frac{k}{C}\right)•k_{B}=\Delta S^{*}$$

The transition enthalpy $\Delta H^{*}$ is considered to be constant and the Arrhenius constant is set to be one so that:

(6) $$\Delta S^{*}\propto k_{B}\ln(k)$$

Equation 6 allows assuming the amount of entropy change ΔS* and its influence on the mechanism of phase transformation. Since the molecules are isotropically disordered in the ME phase and aligned in the liquid phase one might believe that the entropy should decrease. It increases instead, since in an oriented condition the available space for any molecule per volume is increased compared to a randomly oriented state. The loss of the orientational entropy is overcome by a gain in translational entropy of the systems.

The Johnson-Mehl-Avrami-Erofeèv-Kolmogorov (JMAEK) theory is widely used to model a phase transition involving a nucleation and growth mechanism under isothermal conditions. In the following we apply this theory to the ME→LC phase transition. The JMAEK model describes the accelerated growth of LC nuclei which are randomly distributed. The growth consuming the ME phase follows a statistical slowdown of the conversion due to the impingement of the LC nuclei surface. Mathematically this process is described as:

(7) $$y=1-e^{-kt^{n}}$$

where *y* is the fraction of the LC phase, *t* is the time and *k* is the growth constant [12]. The Avrami exponent *n* describes the dimension of the growth in space. For a homogeneous phase transformation in which the probability of the transformation is equal for any region within the sample, *n* = 1. For a one-dimensional growth, *n* = 2, for a two-dimensional growth *n* = 3 and for three-dimensional growth *n* = 4 [12]. Table 1 summarizes the results of the JMAEK-model applied to the kinetics of the investigated systems.

**Table 1:** Kinetic parameters for the ME→LC phase transition. The definitions are given in the text.

C_i_E_4_	*τ* / min	Avrami -exponent *n*	*α* / min^-1^	*β* / min^-2^
8	5.9 ± 0.1	3.6 ± 0.1	0.27 ± 0.08	0.0009 ± 0.0003
10	25 ± 2	1.8 ± 0.1	0.038 ± 0.002	0.0020 ± 0.0003
12	33 ± 2	1.32 ± 0.07	0.0284 ± 0.0008	0.0027 ± 0.0004
14	79 ± 5		-	-

The interpretation of the sigmoidal conversion versus time is not straightforward, since the obtained parameters are not integers and do not match well any of the three values. The Avrami exponent, determined for C_12_E_4_ is *n* = 1.3 leading to a zero-dimensional growing mechanism, which means a homogeneous, star like formation of the LC germs. Moreover, this non-integer Avrami parameter suggests that the growth of the continuously formed nuclei is not perfectly n-dimensional and follows no constant rate through the transformation. In order to improve the models, Skradla has developed an alternative description (homogeneous dispersive kinetics model) for the treatment of nucleation or denucleation processes [13]. According to his model the use of a single activation energy in nucleation based processes is not appropriate. In the homogeneous dispersive kinetics model, the dispersion in the activation energy barrier produces a statistical distribution of activation energies caused by Brownian molecular motion. Considering this leads to a zero-point corrected reaction energy barrier, *E_0_*, which also comprises small changes along the reaction coordinate. It is hypothesized that these small energy differences are part of a statistical distribution produced by various quantum state differences that exist between the activation complex and the reagent species [14]. The form of the Maxwell-Boltzmann distribution can be taken to describe the quantitized energy differences. This distribution of activation energies forms the basis of a distribution of molecular rate constants which can be expressed as time-dependence of the rate constant for the overall transformation. For isothermal homogeneous processes with decelerated velocities, the transformation rate constant decreases as time proceeds according to:

(8) $$y=1-\exp\;\{[-\alpha \;t]\;[\exp(-\beta \;t^{2})-1]\}$$

**Figure 10:**
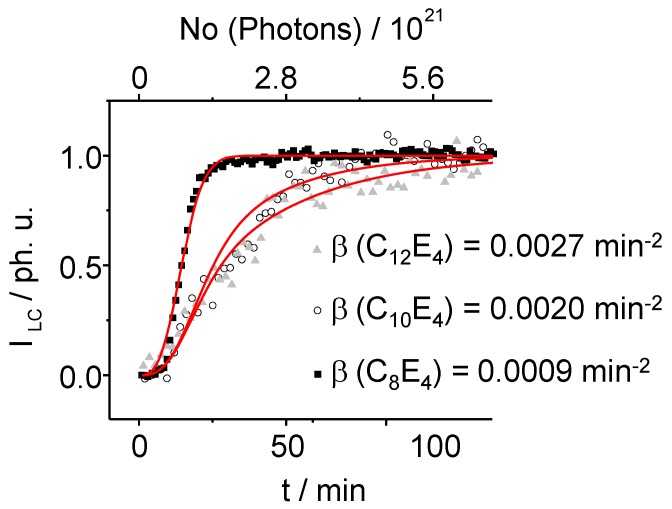
Overlay of the transformation–profile showing the time- dependence of the integral intensity scatter of the LC reflection. The curves are fitted according to the homogeneous dispersive kinetics theory (Equation 8) allowing extracting parameters to determine the transition-entropy (Equation 9). On the basis of this theory, the conversion depends on the value of the time-dependent rate constant.

Here, *y* is the mole fraction of the LC phase formed at time *t*. *α* and *β* are semi-empirical parameters called “global first order” and “global deceleratory“ rate constants with units of time^-1^ and time^-2^ [14]. Note that for an infinity time *α = -k* from Equation (4). In that sense α describes the growing kinetics of the newly formed LC phase and *β* expresses its delays due to an induction and deceleration process. Figure 10 displays the time-dependence of the LC reflection which is fitted according to the homogeneous dispersive kinetics model showing a good compliance between model and the experimental data. It can be derived that *β*, which can be either positive or negative, is linked to the activation entropy of the transformation as:

(9) $$\beta =\frac{\Delta S^{*}(T,t)}{R\;t^{2}}$$

Typical for the growing kinetics of the PIPT is its asymmetric sigmoidal form. The activation energy *E_A_* depends on the transformation time according to:

(10) $$E_{A}=E_{A}^{0}+RT\beta \;t^{2}$$

where $\Delta E_{A}^{0}$ is a fixed potential energy barrier [15]. Taking Equation (4) into account leads to:

(11) $$\Delta G^{*}(T,t)=E_{A}^{0}(T)-2RT=\Delta H^{*}(T)-T\;\Delta S^{*}(T,t)$$

**Figure 11:**
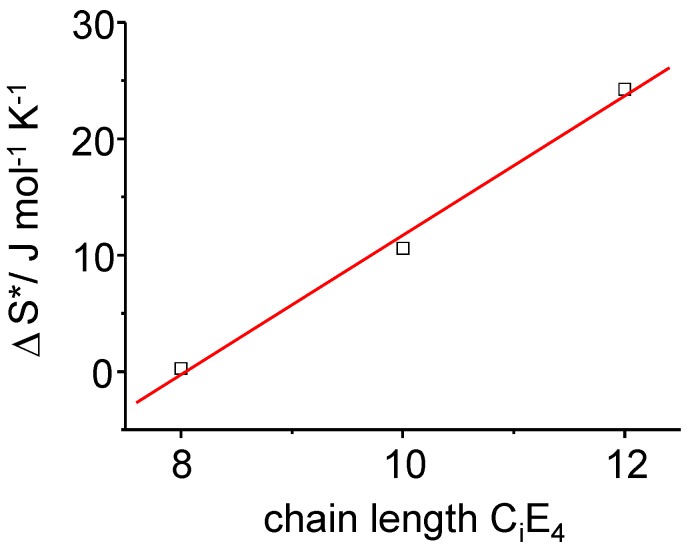
The time-dependent transition entropy ΔS* of the PIPT at a point in time where the transformation is completed to half (τ_1/2_). A linear dependence of ΔS* on the chain length of the surfactants C_i_E_4_ has been found.

Equation (11) demonstrates that *ΔS* is responsible for the time–dependence of the activation energy in dispersive conversions as it relates the kinetic energy redistribution of the initial phase, *i.e.,* the ME phase to the rate of conversion from the ME to the LC phase [13]. In Figure 11, the activation entropy derived from Equation 9 is plotted against the chain length of the C_i_E_4_ systems.

It can clearly be pointed out that the rate differences observed for the three different surfactant systems are driven by the -*T ΔS* *(T, t)* term, which is the entropy term. The increase in *ΔS* results in an increase of *ΔG* (Equation 11) providing ΔH* is constant. For a given temperature it means that the activation energy $\Delta E_{A}^{0}$ increases as time proceeds. So for longer chain lengths, LC formation is inhibited (increase of $\Delta E_{A}^{0}$) due to an increase of diffusional and frictional effects in the longer chain surfactants. These effects can be attributed to molecular motion and diffusion occurring during the phase transformation until the system is again at equilibrium. Hence, prolonging the chain length of the surfactant has an inhibitory effect on the phase transformation. For the overall conversion rate, time-dependent rate constants comprising multiple activation energies, are deviated as:

(12) $$k=2\alpha \beta \exp(-\beta \;t^{2})$$

**Figure 12:**
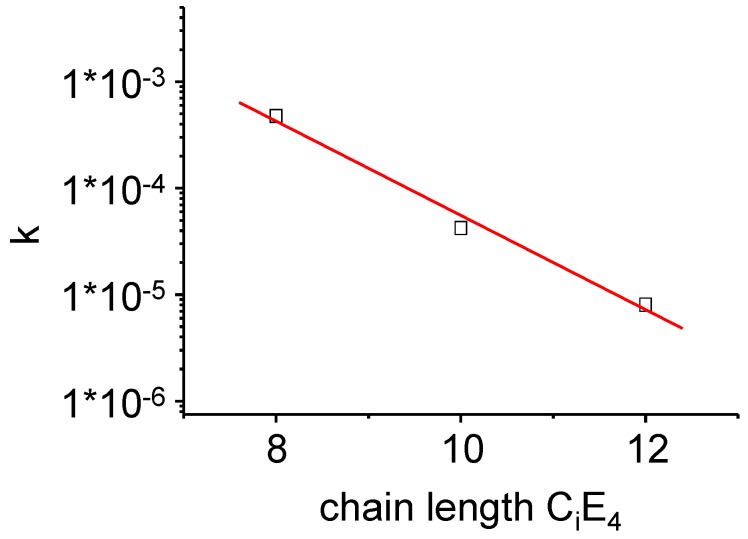
The time-dependent overall conversion rate *k* as defined in Equation (12) of the PIPT at a point in time where the transformation is completed to half (*τ_1/2_*). A lognormal dependence of *k* on the chain length of the surfactants C_i_E_4_ has been found.

Note that Equation (12) is valid for one temperature. Figure 12 shows the overall conversion rate for the three different systems C_8_E_4_, C_10_E_4_ and C_12_E_4_. Again, the increase of activation energy due to the increase of transition entropy results in a decrease of the rate constant and a slowing down of the ME→LC PIPT. The change of the rate constant is caused by the ME→LC concentration fluctuations as the conversion proceeds.

## 3. Experimental Section

### 3.1. Preparation

The systems were prepared as follows: the polyalkyl glycol ethers (surfactants) C_8_E_4_, C_10_E_4_, C_12_E_4_ (98%) were purchased from Fluka and C_14_E_4_ (99%) from Sigma-Aldrich no further purification was done. The alkane series [*n*-decane (98%), *n*-octane (99.5%), *n*-dodecane (98%), *n*-tetradecane (99%)] was obtained from Fluka as well. The surfactants (9.79 × 10^-4^ M), the corresponding alkane (1.07 × 10^‑3^ M) bidistilled water (polar solvent, 1.94 × 10^-2^ M) and cyclohexane (2.70 × 10^-3^ M, 99%, Merck) were mixed for 5 min and subsequently acidified with two drops of fuming hydrochloric acid (37%, Merck). The dye Rh101, (99%, c ~ 2 × 10^-5^ M, Lambda Physics) was added to these mixtures without further purification. The mixture was stirred at room temperature for 1 hour in a thermostat.

The temperatures for the microemulsion–Lα phase transition were determined by using the polarized screening technique (PST) in a thermostatted bath with the solutions of 2 mL contained in sealed test tubes with a variance of ca ± 0.02 °C degrees. The phase transition appears with a clear change on the polarized scattering light and shows a hysteresis of about 2 °C. Since the phase transition appeared relatively fast and with a clear separation of the phases the Lever rule was not used.

### 3.2. Thermodynamic and optical properties

**Figure 13:**
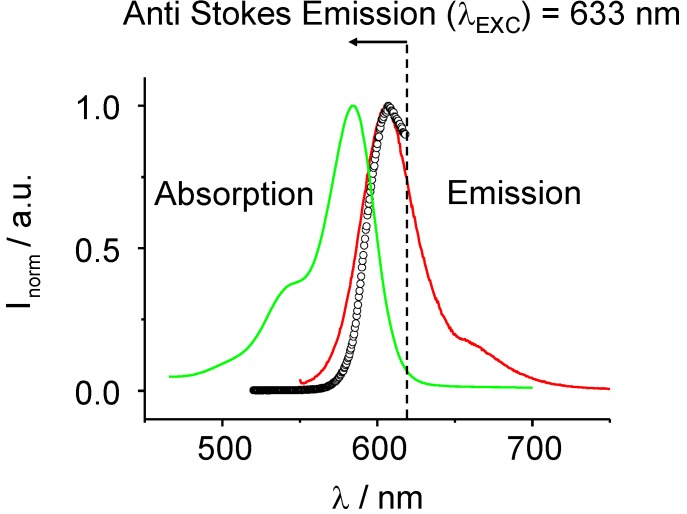
Absorption and fluorescence emission spectra of Rh101 dye-sensitized C_10_E_4_ / water / decane / cyclohexane ternary liquid crystal system. Optical Stokes and Anti-Stokes excitation are marked and shown. The Anti-Stokes excitation has been used to photoinduce the ME→LC phase transition.

The phase diagram is defined by two variables, the temperature *T* and the surfactant concentration with respect to the water and oil concentration. Since the concentration ratios have been kept constant the phase boundary microemulsion-Lα phase decreases almost linearly with an increase of the chain length of the surfactant. The following transition-temperatures have been measured: C_8_E_4_ (23.7 °C), C_10_E_4_ (19.4 °C), C_12_E_4_ (12.1 °C) and C_14_E_4_ (7.1 °C). Figure 13 confirms the optical properties of the Rh101 dye in the ME phase (normalized absorption and emission spectra). The optical excitation conditions were chosen in such a way that the ratio of chromophore to optically exciting photons is about 10,000:1. The used optical conditions were chosen to be in the linear regime yielding slow transformation times. The dye was anti-Stokes excited at 632.8 nm (with respect to the dyes fluorescence maximum at about 608 nm / 16450 cm^-1^, Figure 13).

Quantum yield measurements verified that the Rh101 fluorescence quantum yield is still 1.0 in the microemulsion phase indicating that the dye’s local vicinity is polar and most probably the water volume part in the microemulsion phase.

### 3.3. Absorption and fluorescence spectroscopy

Absorption spectra were recorded using a Cary-5E UV-VIS-NIR spectrophotometer (Varian Australia Pty Ltd) and fluorescence spectra with a Fluorolog 3-22 (Jobin Yvon-Spex, Munich). Detailed description of the experimental conditions can be found elsewhere [8].

### 3.4. Time-resolved small angle x-ray scattering (SAXS)

In contrast to the TR-SAXS apparatus described recently, in the current setup the detector was read out by the PC-based, software controlled PCI-bus multichannel analyzer MCA-FADC with 500 ns conversion time from Fast ComTec [6]. With this setup, a scattering pattern was measured every 30 s. The whole experimental procedure was automatised.

The laser beam was widened in order to obtain an ellipsoidal spot of about 5 mm^2^. The probing area, defined by the slitwidth of the Kratky-camera, was 3 mm^2^ in order to assure a good spatial overlap. Spatial averaging for the change induced by the pumping is therefore excluded effectively.

## 4. Conclusions

In summary, we have shown that the effect of the chain length on the transformation rate of the ME→LC PIPT is significant though the phase transition is initialized by optical photons. The rate constant increases linearly from τ _LC_ = 4 min for C_8_E_4_ to τ _LC_ = 77 min for C_14_E_4_. We attribute this to the time needed for the ordering of the alkyl chains. The longer the chain, the longer the ordering time until the molecule fits properly into the liquid crystal lattice, allowing it to arrange. This is also reflected by the entropy dependence of the chain length as has been derived from the kinetic data. The overall growth rate is slightly reduced by the mass transport since an increase in viscosity leads to a decrease in diffusivity [16]. While more and more molecules attach, the periodic distance between them shrinks monoexponentially with time. This effect is more pronounced in systems with longer chain lengths, for instance 3% in C_12_E_4_, than in those with shorter chains like in C_8_E_4_ (0.7%) and can be explained with a rearrangement in form of a shearing motion and straighter alignment of the oriented molecules in respect to each other. The restructuring of the PIPT-formed phase on this long time scale leads to a higher order in the lamellar stacking in the case of the ME→LC PIPT.

Therefore the dynamics of the PIPT is photo-induced, the absorption of the photons give the first energy-“kick” for overcoming the activation energy to form seeds. However – after this first initial step - the seed growing kinetics is determined by diffusion processes and entropy effects.
